# Pharmacy Customers’ Experiences of Use, Usability, and Satisfaction of a Nationwide Patient Portal: Survey Study

**DOI:** 10.2196/25368

**Published:** 2021-07-16

**Authors:** Maria Sääskilahti, Riitta Ahonen, Johanna Timonen

**Affiliations:** 1 School of Pharmacy Faculty of Health Sciences University of Eastern Finland Kuopio Finland

**Keywords:** patient portal, electronic prescription, electronic health records, usability, pharmacy customer, perception, experience, survey

## Abstract

**Background:**

Patient portals have been introduced in several countries in the last few decades. Despite worldwide objectives of introducing patient portals, nationwide portals are rare, and studies about their use are limited. Finland is one of the forerunners in developing nationwide health data systems. A nationwide patient portal, My Kanta, for viewing electronic prescriptions and health data has been phased in, starting in 2010.

**Objective:**

The aim of this study was to investigate what functions Finnish pharmacy customers use in My Kanta, their perceptions of the service’s usability, and how satisfied users are with My Kanta overall.

**Methods:**

In spring 2019, questionnaires (N=2866) were distributed from 18 pharmacies of varying size across mainland Finland to adult pharmacy customers purchasing prescription medications for themselves or for their child under 18 years of age. Questions were asked about the use and usability of the patient portal by means of structured and Likert-scale questions. Statistical analyses included frequencies, means, medians, chi-square tests, Fisher exact tests, and Kruskal-Wallis tests.

**Results:**

In total, 994 completed questionnaires of 2866 delivered questionnaires (34.68%) were returned. The most-used My Kanta functions were browsing prescription information (781/802, 97.4%), records of health care visits (772/802, 96.3%), and results of laboratory tests and x-ray examinations (722/804, 89.8%). Most users (558/793, 70.4%) had also requested a prescription renewal using the service. My Kanta was perceived as easy to log into (772/816, 94.6%) and clear to view (709/808, 87.7%). Most users considered the service useful for monitoring their health information (753/813, 92.6%) and felt that it provides a good overall picture of the medications prescribed to them (711/813, 87.5%). The majority of users found the information recorded about them easy to understand (684/800, 85.5%). Of the users, 16.7% (135/807) disagreed with the statement that the information they were looking for was easy to find. Approximately two-thirds (501/814, 61.5%) of users did not know whether it is easy to view in which pharmacies and health care units their prescription information has been viewed, and over one-third (306/805, 38.0%) did not know whether it is easy to view in which health care units their health information has been processed. Approximately one-fifth of participants (181/805, 22.5%) feared that unauthorized persons might view their information and that their electronically saved prescription and health information might disappear (180/810, 22.2%). In addition, 16.1% (129/799) expressed interest in receiving guidance on My Kanta use. The vast majority of users (719/804, 89.4%) were satisfied with the service overall.

**Conclusions:**

Pharmacy customers were satisfied with the nationwide patient portal. It was mostly used for browsing e-prescriptions and medical records. Overall, the usability of the service was good. However, users need to be better informed about data privacy and security issues, and guidance on using the portal needs to be improved.

## Introduction

### Background

Patient portals displaying electronic health records have been developed worldwide in the last few decades. One aim of patient portals is to increase patients’ empowerment and their responsibility for their own health and well-being [1-6]. In most countries, for example, in the United States, the Netherlands, and the United Kingdom, portals cater to patients of a specific organization, with a particular disease, or of a specific region [3,5,7-11], while nationwide portals are rare. Nationwide portals have, however, been introduced in the Nordic countries and Estonia [12-18].

The contents and functions of portals vary between countries and portals. Portals based on an organization, disease, or region often include functions such as viewing medical notes, visit summaries, diagnoses, laboratory tests, and medications; scheduling appointments; renewing prescriptions; and secure messaging with health care professionals [3,7-10]. The contents of nationwide portals are broadly the same as in organization-based or disease-specific portals, allowing patients to monitor health and medication data [2,12-15,17,18]. However, nationwide portals seldom include the opportunity to request a prescription renewal, schedule appointments, or communicate with health care professionals. On the other hand, some nationwide portals allow users to declare organ donation testaments, access log lists (a list where one can view who or which organization has viewed one’s information), or restrict access to health records. Nationwide patient portals also vary in terms of how widely available the health records are from different units (eg, hospitals, private providers, or health centers) or regions. For example, some private providers in Sweden and some regions in Norway do not provide access to their records, whereas electronic health records from all units in Denmark and Finland are available to patients.

Studies about the use and patients’ perceptions of organization-based or disease-specific portals are common, most of them conducted in the United States and the Netherlands. Studies show that the most-used functions of patient portals are viewing laboratory results and medical records [3,5,7-10]. Studies on the usability of services have shown that, overall, patients are satisfied with the services [3,8,10,19,20]. However, there are few studies about the use and usability of nationwide patient portals [13,14,17]. The World Health Organization is encouraging its member states to develop national digital health systems [21]. It is thus important to study the use and usability of nationwide portals to provide countries developing such systems information to support their development work. In addition, users’ perceptions of portal use are also important to help make existing systems more useful and user-friendly.

In Finland, a nationwide patient portal, My Kanta, has been introduced. The aims of this study were to investigate what functions are used in My Kanta, users’ perceptions of the service’s usability, and how satisfied users are with My Kanta overall.

### Study Context

In Finland, Kanta services are nationwide digital health care and social welfare services intended to be used by health care professionals, pharmacies, and citizens throughout the country [22]. The services have been phased-in starting in 2010 and are continuously under development. Kanta services are maintained and developed in cooperation with national authors such as the Social Insurance Institution of Finland, the Ministry of Social Affairs and Health, and the National Institute for Health and Welfare [23].

In Finland, it has been obligatory to issue all prescriptions electronically since the beginning of 2017 [24]. Every stage of the Finnish prescribing system is electronic. A physician saves electronic prescriptions (e-prescriptions) to a centralized database, called the Prescription Centre, from where they can be retrieved for dispensing in every pharmacy across Finland. In exceptional situations, such as power blackouts, paper or telephone prescriptions are permitted, but conventional prescriptions are saved to the Prescription Centre at pharmacies when dispensing the prescription for the first time. All Finnish public health care units (primary and special health care) and private health care providers record patients’ health care data in the nationwide Patient Data Repository [25]. Health data has been recorded in this service from public health care since 2013 and from private units since 2016. To access the Kanta services, health care and pharmacy professionals need to verify their identity with strong electronic identification (a smart card). All data viewing and processing by professionals can be traced.

Prescriptions and health data recorded in one unit can be shared with other units, with the patient’s consent [25]. e-Prescriptions can be viewed in pharmacies and health care units via oral consent from the patient. To share health data, informed consent from the patient has to be approved and saved in Kanta services. The consent is valid until further notice and covers all the health data recorded in the Patient Data Repository. However, the patient can deny the sharing of certain health data (eg, certain health care visits or all the data of certain units) or e-prescriptions. All consents and refusals can be approved or canceled by the patient in health care units or in My Kanta.

My Kanta, a part of Kanta services, is an online service allowing information about e-prescriptions and health data to be viewed by patients [26]. Every person with a Finnish identity number and an ID for electronic services, such as an online banking code, can sign into the service. My Kanta shows an overview of the user’s e-prescriptions: when and where the prescription was issued, name of the prescriber, dosage instructions, valid date of prescription, whether there is any medication left, when and where the medication was purchased, and whether the prescription has been renewed. Health data shown in the service consist of records of health care visits, diagnoses, critical risk factors, laboratory tests, x-ray examinations, referrals, health and care plans, and medical certificates and statements (issued, for example, to secure allowances from the Finnish Social Insurance Institute). Using My Kanta, patients can request a prescription renewal, print out a summary of their e-prescriptions, consent to or limit the disclosure of personal data, record living wills and organ donation testaments, and view in which health care units and pharmacies their personal data has been viewed or processed (later referred to as browsing disclosed information). Guardians can view the health data and e-prescriptions of dependents under 10 years of age and also request a renewal of dependents’ prescriptions. This paper focused on functions concerning participants’ personal data.

## Methods

### Data Collection

In spring 2019, a questionnaire survey was conducted among pharmacy customers aged 18 years or older who were purchasing prescription medications for themselves or for their child under 18 years of age. Questionnaires (Multimedia Appendix 1) were distributed by 18 community pharmacies of varying size across mainland Finland (of 623 total pharmacies in Finland). Pharmacies were recruited from all 6 Regional State Administrative Agency areas in mainland Finland. One university pharmacy branch (owned by a university but operating as a privately owned pharmacy); one large, privately owned, urban pharmacy; and one small, privately owned, rural pharmacy were chosen from each region using convenience sampling. Pharmacies were instructed to offer questionnaires to all eligible customers after dispensing prescription medications. The number of questionnaires delivered to a pharmacy was in relation to the number of prescriptions dispensed annually at that pharmacy and varied between 40 and 320 questionnaires. In total, 3560 questionnaires were delivered to the pharmacies. Pharmacists requested customers to complete the questionnaires at home and to post them in return envelopes to the research group. Pharmacies did not keep a record of customers taking questionnaires or refusing to participate in the study. Pharmacies distributed questionnaires for a maximum of 2 weeks. After the study period, pharmacies informed the research group of how many questionnaires remained, to allow the response rate to be calculated. In total, 2866 questionnaires were distributed.

### Questionnaire

The questionnaire was designed based on My Kanta pages and previous studies about patient portals [3-5,16,19,26-29]. It was tested for face validity by 3 researchers experienced with designing questionnaires, before a pilot test at a pharmacy. In the pilot test, pharmacy customers completed questionnaires and discussed the questions and their intelligibility with researchers. Minor revisions were made as a result.

The questionnaire included 22 questions and was divided into 3 parts. The first part was for all participants and concerned background information, the second was for users of the service, and the third part was for those who did not use the service. Questions about background information (ie, gender, age, education, region, internet use, internet use for searching health-related information, existence of chronic diseases, and number of currently used, regular prescriptions) were structured except for age and number of currently used, regular prescriptions, which took the form of open-ended questions.

This paper reports results from 4 of the questions from the second part of the questionnaire. Two structured questions concerned the use of different functions in My Kanta, asking “Have you used the following functions in My Kanta?” The first question concerned e-prescriptions and health data, and response options were “often,” “sometimes,” “rarely,” and “never.” The second question concerned consenting and limiting consent, for which the response options were “yes,” “no,” and “do not know.” A 5-point Likert-scale question, *“*What do you think about the following statements?*”* with response options “fully agree,” “agree to some extent,” “disagree to some extent,” “fully disagree,” and “do not know,” included 18 statements about the service and its usability. A 6-point Likert-scale question concerned users’ overall satisfaction with My Kanta: “How satisfied are you with My Kanta as a whole?” with responses ranging from 1 (“not satisfied at all”) to 6 (“very satisfied”).

### Statistical Analysis

Statistical analyses were conducted using SPSS software (version 25.0; IBM Corp). Descriptive analyses included frequencies, means, and medians. Differences in the use of My Kanta functions between participants were examined using the chi-square test and Fisher exact test. The nonparametric Kruskal-Wallis test was used to analyze differences between means in independent groups for satisfaction with My Kanta. Statistical significance was determined as *P*<.05.

For the analyses, participants’ years of birth were converted to ages, in years, and then categorized into 4 age groups: 18-34, 35-59, 60-74, and ≥75. In the questionnaire, education had 5 response options: “basic education,” “vocational degree,” “secondary school graduate,” “lower university degree,” and “higher university degree.” For the analyses, “vocational degree” and “secondary school graduate” were combined into “secondary education,” and “lower university degree” and “higher university degree” were combined into “university degree.” In the question concerning existence of chronic diseases, the responses “do not know” were regarded as missing values due to the low number of these responses. The number of regularly used prescription medications was placed into 3 groups: 0, 1-4, and ≥5. In the Likert-scale question about users’ perceptions of My Kanta and its usability, response options “fully agree” and “agree to some extent” were combined, and “fully disagree” and “disagree to some extent” were also combined.

### Ethical Statement

According to the National Instruction for Research Ethics [30], this study did not require ethical approval. However, approval was obtained at the request of the funding organization from the Committee on Research Ethics of the University of Eastern Finland (number 23/2018). Participation in the study was voluntary; responding to the questionnaire and posting it to the researchers was regarded as informed consent to participate. No incentives were provided for participating in the study. Pharmacy owners permitted the distribution of questionnaires at their pharmacies.

## Results

### Study Population

In total, 996 questionnaires were returned. Two of them were blank and were therefore excluded from the study. The final study sample comprised 994 responses from the 2866 questionnaires distributed (34.68%). Over two-thirds (687/990, 69.4%) of participants were female (Table 1), and the mean age was 62 years (range 18-99, median 66). Participants were from all 6 regions across Finland. Of all participants, 82.5% (820/994) were My Kanta users. The characteristics of My Kanta users were very similar to those of all participants except for more frequent internet use and internet use for searching health-related information. In addition, there appears to be less participants aged 75 years or older among My Kanta users.

**Table 1:** Study population characteristics.

Variable		Total participants (N=994)^a^, n (%)	My Kanta users (n=820)^a^, n (%)
**Gender**		990	819
	Female	687 (69.4)	576 (70.3)
	Male	303 (30.6)	243 (29.7)
**Age (years)**		958	791
	18-34	54 (5.6)	50 (6.3)
	35-59	269 (28.1)	236 (29.8)
	60-74	467 (48.7)	396 (50.1)
	≥75	168 (17.5)	109 (13.8)
**Education**		994	820
	Basic education	185 (18.6)	129 (15.7)
	Secondary education	523 (52.6)	444 (54.1)
	University degree	286 (28.8)	247 (30.1)
**Region**		992	818
	Southern Finland	135 (13.6)	107 (13.1)
	Southwestern Finland	144 (14.5)	109 (13.3)
	Western and Central Finland	192 (19.4)	155 (18.9)
	Eastern Finland	224 (22.6)	189 (23.1)
	Northern Finland	222 (22.4)	193 (23.6)
	Lapland	75 (7.6)	65 (7.9)
**Internet use**		987	814
	Daily or on several days a week	851 (86.2)	772 (94.8)
	Once a week or less often	79 (8.0)	42 (5.2)
	Not at all	57 (5.8)	0 (0.0)
**Internet use for searching health-related information**		991	819
	Yes	842 (85.0)	770 (94.0)
	No	149 (15.0)	49 (6.0)
**Has any chronic disease diagnosed by a physician**		982	809
	Yes	823 (83.8)	682 (84.3)
	No	140 (14.3)	113 (14.0)
	Does not know	19 (1.9)	14 (1.7)
**Current use of regular prescription medications**		942	780
	0	101 (10.7)	87 (11.2)
	1-4	604 (64.1)	496 (63.6)
	≥5	237 (25.2)	197 (25.3)
**Use of the My Kanta service**		994	820
	Yes	820 (82.5)	820 (100.0)
	Has used it but is not going to use it anymore	21 (2.1)	0 (0.0)
	Has never used it	153 (15.4)	0 (0.0)

^a^Some participants did not answer the question. Therefore, the total for each variable category differs.

### Use of Different Functions in My Kanta

#### Use of Functions Concerning e-Prescriptions and Health Data

**Figure 1:**
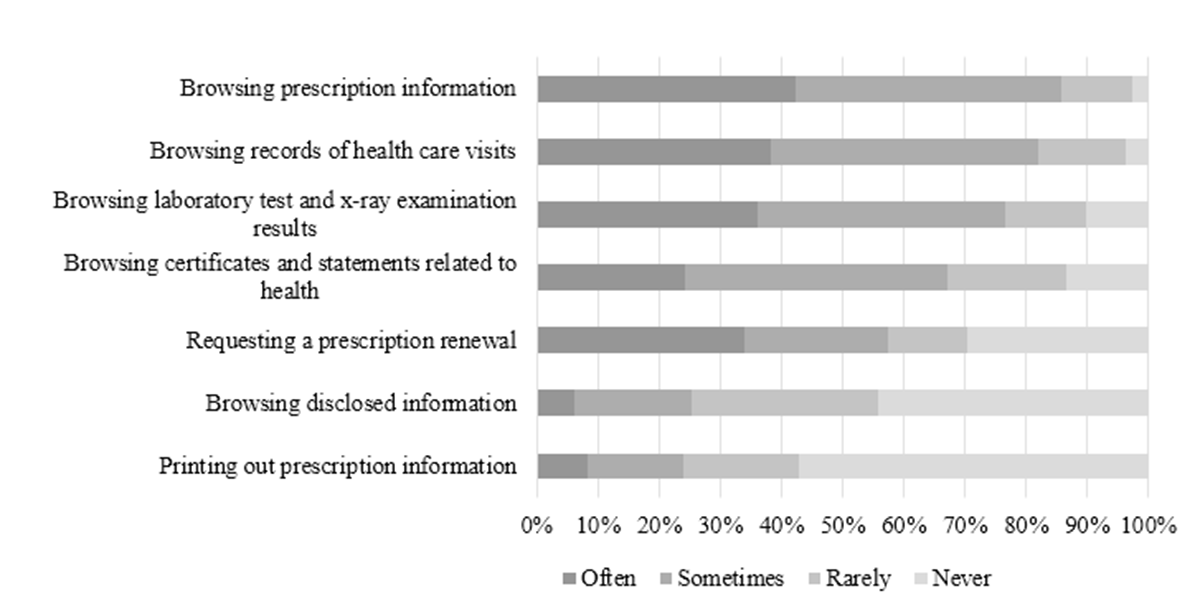
Frequency of using functions concerning electronic prescriptions and health data in My Kanta.

The most-used My Kanta functions concerning e-prescriptions and health data were browsing prescription information (781/802, 97.4%), records of health care visits (772/802, 96.3%), and results of laboratory tests and x-ray examinations (722/804, 89.8%) (Figure 1). Over one-third of users used these functions often (340/802, 42.4%; 306/802, 38.2%; 290/804, 36.1%; respectively). Browsing prescription information was associated with internet use (*P*<.001); use of the internet to search for health-related information (*P*<.001); existence of chronic diseases (*P*<.001); and number of currently used, regular prescription medications (*P*<.001) (Multimedia Appendix 2). Records of health care visits was also associated with internet use (*P*=.002); use of the internet to search for health-related information (*P*<.001); existence of chronic diseases (*P*=.02); and number of currently used, regular prescription medications (*P*=.003) . For example, those who seldom used the internet or did not use the internet to search for health-related information more commonly had never browsed prescription information or records of health care visits. Users without chronic diseases were less likely to have often browsed prescription information or records of health care visits. Users who currently used ≥5 regular prescription medications more commonly had often browsed prescription information and records of health care visits.

Of My Kanta users, 70.4% (558/793) had requested a renewal of their prescriptions in the service (Figure 1). Approximately one-third did this often (268/793, 33.8%). Requesting a prescription renewal was associated with education (*P*=.01), internet use (*P*=.01), existence of chronic diseases (*P*<.001), and number of currently used, regular prescription medications (*P*<.001) (Multimedia Appendix 2). For example, users with a university degree, those who seldom used the internet, users without chronic diseases, or those who did not use any regular prescription medications more commonly had never requested a prescription renewal. Instead, those who currently used ≥5 regular prescription medications more commonly had often requested a prescription renewal in My Kanta.

The least-used functions were browsing disclosed information (438/787, 55.7%) and printing out prescription information (327/765, 42.7%) (Figure 1). Users with a basic education more commonly browsed disclosed information often, whereas users with a university degree more commonly had never browsed disclosed information (*P*=.001) (Multimedia Appendix 2). Printing out prescription information was associated with gender (*P*=.02), age (*P*=.001), existence of chronic diseases (*P*<.001), and number of currently used, regular prescription medications (*P*<.001). For example, men less often had never printed out prescription information. Users aged 75 years and older or those who currently used ≥5 regular prescription medications more commonly printed out prescription information often. Users without chronic diseases more commonly had never printed out prescription information.

#### Consents and Limitations

**Figure 2:**
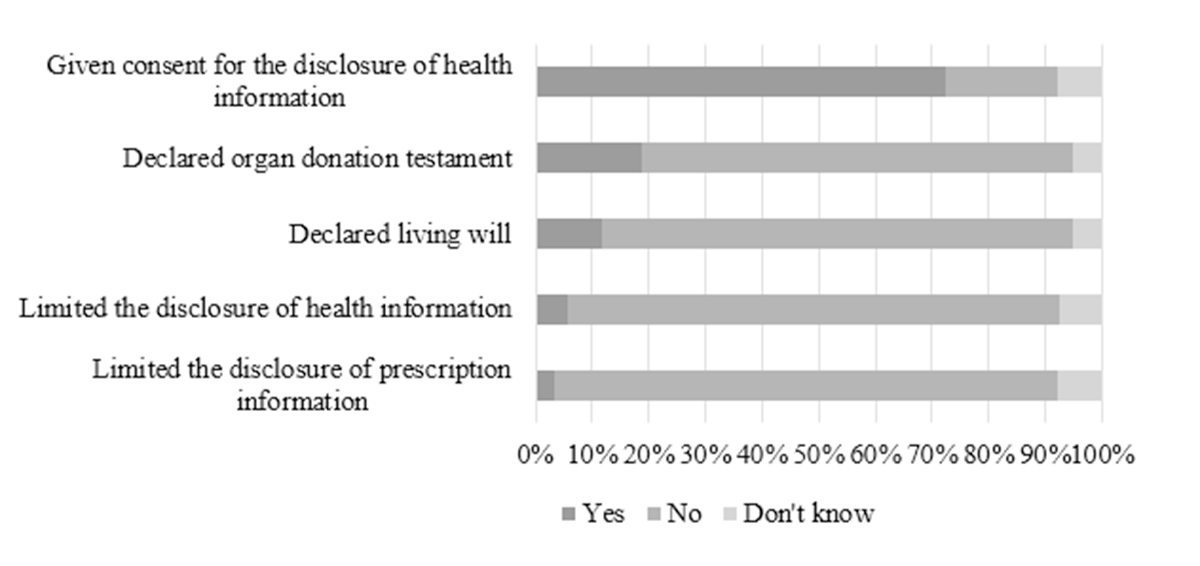
Use of functions concerning consents and limitations in My Kanta.

Most My Kanta users (584/808, 72.3%) had consented to disclosure of their health information in the service (Figure 2). Approximately one-fifth of users (151/800, 18.9%) had declared an organ donation testament and approximately one-tenth (95/803, 11.8%) had declared a living will in the service. It was rare that participants limited disclosure of health (45/797, 5.6%) and prescription information (26/799, 3.3%). Giving consent for disclosure of health information was associated with age (*P*=.001) and internet use (*P*<.001) (Multimedia Appendix 3). Young participants (18-34 years), more commonly than older participants, did not know whether they had given consent for disclosure of health information. In addition, those who seldom used the internet had less often consented to disclosure of health information in the service. Age was associated with limiting the disclosure of health (*P*=.02) and prescription information (*P*=.04). For example, young participants (18-34 years), more commonly than older participants, did not know whether they had limited the disclosure of health data. Declaring an organ donation testament was associated with age (*P*<.001), education (*P*<.001), and internet use (*P*=.03). For example, participants aged 18-59 years had more commonly declared an organ donation testament, whereas those 75 years and older had declared it less often. In addition, those with a basic education had declared an organ donation testament less often, while the declaration was more common among participants with a university degree.

### Usability of My Kanta

**Figure 3:**
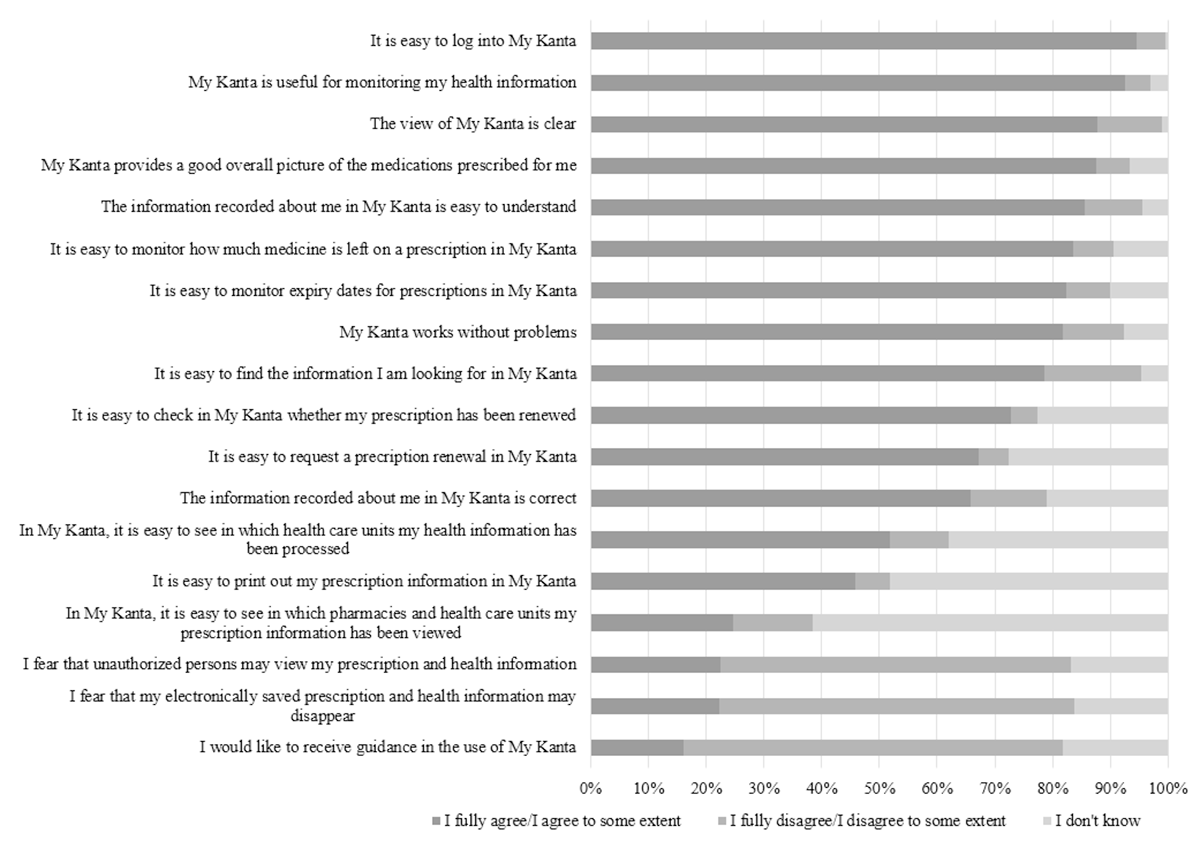
Participants’ perceptions of My Kanta and its usability.

Most My Kanta users reported that My Kanta is easy to log into (772/816, 94.6%), its view is clear (709/808, 87.7%), it works without problems (655/802, 81.7%), and it is easy to find the information they are looking for (634/807, 78.6%) (Figure 3).

Most users said My Kanta is useful for monitoring their health information (753/813, 92.6%) and that the service provides a good overall picture of the medications prescribed to them (711/813, 87.5%) (Figure 3). A high proportion of users agreed that, in My Kanta, it is easy to monitor how much medicine is left for a prescription (682/817, 83.5%) and what the expiry dates for prescriptions are (668/811, 82.4%). Most users found the information recorded about them easy to understand (684/800, 85.5%).

Conversely, 16.7% (135/807) of users disagreed with the statement that it is easy to find the information they are looking for (Figure 3). Approximately two-thirds (501/814, 61.5%) of users did not know whether it is easy to view in which pharmacies and health care units their prescription information has been viewed. In addition, over one-third (306/805, 38.0%) did not know whether it is easy to view in which health care units their health information has been processed. Approximately one-fifth of participants feared that unauthorized persons might view their prescription and health information (181/805, 22.5%) and that their electronically saved prescription and health information might disappear (180/810, 22.2%). Altogether, 16.1% (129/799) of My Kanta users expressed interest in receiving guidance on using the service.

### Overall Satisfaction With My Kanta

On the 6-point Likert scale, 89.4% (719/804) of My Kanta users had rated their overall satisfaction with the service within a range of 4-6 (mean 4.8; median 5) (Table 2). Overall satisfaction differed significantly across participants’ education levels (*P*=.03) and frequency of internet use (*P*=.001). Participants with a basic education were more satisfied with My Kanta than those with a university degree, and participants who used the internet daily or several times a week were more satisfied than those who used the internet once a week or less.

**Table 2:** Participants’ overall satisfaction rating^a^ with My Kanta (N=804).

Participant group		Rating							*P* value
		1, n (%)	2, n (%)	3, n (%)	4, n (%)	5, n (%)	6, n (%)		
All participants		7 (0.9)	16 (2.0)	62 (7.7)	153 (19.0)	373 (46.4)	193 (24.0)	N/A^b^	
**Gender**									.11
	Female	5 (0.9)	13 (2.3)	42 (7.4)	118 (20.9)	256 (45.4)	130 (23.0)		
	Male	2 (0.8)	3 (1.3)	20 (8.4)	35 (14.6)	116 (48.5)	63 (26.4)		
**Age (years)**									.33
	18-34	0 (0.0)	0 (0.0)	0 (0.0)	10 (20.0)	29 (58.0)	11 (22.0)		
	35-59	0 (0.0)	2 (0.9)	16 (6.9)	51 (22.0)	105 (45.3)	58 (25.0)		
	60-74	4 (1.0)	11 (2.8)	35 (9.0)	64 (16.5)	187 (48.1)	88 (22.6)		
	≥75	3 (2.9)	3 (2.9)	10 (9.6)	23 (22.1)	41 (39.4)	24 (23.1)		
**Education**									.03
	Basic education	3 (2.3)	2 (1.6)	5 (3.9)	21 (16.4)	54 (42.2)	43 (33.6)		
	Secondary education	3 (0.7)	9 (2.1)	36 (8.3)	79 (18.3)	205 (47.5)	100 (23.1)		
	University degree	1 (0.4)	5 (2.0)	21 (8.6)	53 (21.7)	114 (46.7)	50 (20.5)		
**Internet use**									.001
	Daily or on several days a week	5 (0.7)	12 (1.6)	55 (7.2)	142 (18.7)	357 (47.0)	188 (24.8)		
	Once a week or less often	1 (2.6)	2 (5.1)	7 (17.9)	10 (25.6)	14 (35.9)	5 (12.8)		
**Internet use for searching health-related information**									.06
	Yes	5 (0.7)	15 (2.0)	56 (7.4)	142 (18.8)	354 (46.8)	184 (24.3)		
	No	2 (4.3)	1 (2.1)	6 (12.8)	11 (23.4)	18 (38.3)	9 (19.1)		
**Has any chronic disease diagnosed by a physician**									.18
	Yes	5 (0.7)	16 (2.4)	53 (7.9)	131 (19.6)	306 (45.9)	156 (23.4)		
	No	2 (1.8)	0 (0.0)	8 (7.1)	19 (17.0)	51 (45.5)	32 (28.6)		
**Current use of regular prescription medications**									.54
	0	1 (1.2)	1 (1.2)	6 (7.0)	25 (29.1)	33 (38.4)	20 (23.3)		
	1-4	4 (0.8)	11 (2.3)	37 (7.6)	85 (17.5)	231 (47.5)	118 (24.3)		
	≥5	1 (0.5)	3 (1.6)	15 (7.8)	36 (18.8)	92 (47.9)	45 (23.4)		

^a^Rating scale: 1 (“not satisfied at all”) to 6 (“very satisfied”).

^b^N/A: not applicable.

## Discussion

### Principal Results and Comparison With Prior Work

Finnish pharmacy customers mostly used the nationwide patient portal to browse their e-prescriptions and records of health care visits. e-Prescriptions and My Kanta were introduced in Finland in 2010. Since 2017, all prescriptions have been issued electronically, and My Kanta is the only place where patients themselves can find up-to-date information about their e-prescriptions. The frequent browsing of e-prescriptions is therefore understandable. However, the frequent use of this function is a positive finding as, in a study conducted in 2015 in Finland, pharmacy customers felt that the biggest problem with e-prescriptions was keeping up to date with their medication [31]. In this study, users reported that the patient portal provides a good overall picture of their medication and makes e-prescriptions easy to monitor. These findings, and the fact that portal use has significantly increased in the last few years [32], suggest that people have learned to monitor their prescription information via the online service. Use of the patient portal mainly to view prescriptions differs from the findings of previous studies about patient portals [9,10,13,14,17]. This may be because a fully electronic, nationwide prescribing system integrated into a nationwide electronic health record system is rare worldwide [33], and the use of patient portals to monitor medication is not as essential in other countries.

Although monitoring e-prescriptions via My Kanta has been regarded as easy, almost half of My Kanta users, especially older users and those using several regular prescription medications, have printed out prescription information via the service. This suggests that although most My Kanta users can manage their medication via the online service, for others, the printed information is still necessary.

In addition to e-prescriptions, almost all My Kanta users browsed records of their health care visits and test results via the service. My Kanta was perceived as useful for monitoring health data. This is in line with previous studies, which found viewing medical records and results of laboratory tests to be the most-used or useful functions in patient portals [3,5,7,10,13,14]. In previous studies, health data have mainly been used to prepare for health care visits, to reread medical information after visits, and to become more aware and involved in patients’ own health and care. Future studies should examine the reasons for My Kanta use, in order to find out what role the patient portal has in patients’ involvement in their own health and care.

This study showed no differences in the frequencies of using My Kanta for monitoring health and prescription information between user characteristics (ie, gender, age, and education). Instead, using the internet only seldom and not using the internet to search for health-related information were associated with not using My Kanta for browsing health and prescription information. This suggests that information in patient portals is browsed by those who are generally interested in their own health information and search for it on the internet. In agreement with these results, in a previous study, greater health literacy was associated with the use of a patient portal to check test results, whereas gender, age, and education were not [4].

The vast majority of users had used the service to request a prescription renewal. This function has been available in My Kanta since 2015. According to Kanta services’ statistical reports, the number of prescription renewal requests is continuously increasing [32]. In 2019, approximately 250,000 renewal requests were submitted monthly via My Kanta (of approximately 2.5 million monthly issued e-prescriptions in Finland). A study conducted in Finland in 2019 showed that physicians regard the fact that patients can request a prescription renewal in My Kanta as largely beneficial, and one reason was that it saves nurses time [34]. However, physicians also saw this function as problematic, as patients can send a request for any medications to any health care units across Finland regardless of where the prescription was issued. Physicians also felt that allowing patients to submit their own renewal requests may cause difficulties in pharmacotherapy monitoring, as physicians have to search for all necessary information in support of a renewal. In the future, it will be important to study how patients’ renewal requests have affected physicians’ workloads and pharmacotherapy monitoring and, therefore, medication safety. The opportunity to request a prescription renewal is rare in nationwide patient portals. To the best of our knowledge, the function is available in Denmark and Iceland [2,12], although there are no studies reporting the use of this function.

Declaring an organ donation testament or a living will was a rarely used function in My Kanta. Of countries with a nationwide patient portal, at least in Denmark, Estonia, and Iceland, patients have an opportunity to register an organ donor testament [12,18], but studies on the use of this function are unavailable. The purpose of an organ donation testament or a living will is to help health care professionals and patient’s relatives make decisions relating to care in unexpected situations [35]. In Finland, according to law, the organs of a deceased person can be salvaged to treat other patients unless the deceased had previously declined [36]. My Kanta is an easy way to record an organ donation testament and living will, and, via the service, wills are secure and available for health care professionals in situations where patients are not able to express their will themselves. The significance and importance of expressing one’s will should be clarified for citizens, to increase the use of these functions. This study showed that younger people and people educated with a university degree were more likely to declare an organ donation testament.

According to this study, pharmacy customers have rarely limited the disclosure of e-prescriptions and health data. In Finland, one key aim of e-prescriptions is to improve the management of overall medication [24]. It is important that health care professionals can observe patients’ overall medication whenever needed. Another aim of Kanta services is to enable cooperation between health care units and secure the continuity of care [22]. The findings of this study suggest that patients have not prevented achievement of these aims by limiting data disclosure. However, in previous studies, physicians found it difficult to view patients’ overall medication via the Prescription Centre, as there is no list of currently used medications [34,37]. This problem will be solved in the future, as Kanta services are developing a national medication list where up-to-date information about currently used medications is available [38]. Further studies are needed to investigate how health care professionals experience the usability of shared e-prescriptions and health data when caring for patients.

Pharmacy customers were, overall, satisfied with My Kanta. This is in line with previous studies [3,13,17,39], which showed that users are largely satisfied with the patient portals they are using. Compared to a study conducted in 2015 that investigated viewing e-prescriptions via My Kanta [16], pharmacy customers’ perceptions about the usability of the service have remained broadly the same. This is encouraging, as My Kanta is perceived as easy to sign into and monitor e-prescriptions information, the service works without problems, and its layout is regarded as clear. However, there are also some challenges involved in the use of My Kanta that have remained. For example, a substantial proportion of users still did not know whether it is easy to view in which health care units or pharmacies their information has been viewed and processed. This may indicate that users do not know that this information can be found in the service or that they have not tried to search for it. These assumptions are supported by the finding that almost half of users had never browsed disclosed information in My Kanta. However, some My Kanta users were worried that unauthorized persons might view their information. The situation has not changed since 2015 when the issue was last studied [40]. It is therefore important to inform people and My Kanta users, specifically, about the data protection and privacy procedures in Kanta services [41] and that disclosed information can be checked in My Kanta. This might ease unnecessary concerns about data protection.

Data recorded in My Kanta were mostly perceived as easy to understand. However, approximately one-tenth of users disagreed. This is supported by some previous studies, which revealed that the language used in patient portals is sometimes too complicated for patients and should be simplified [10,17]. In Finland, the patient portal is meant for all citizens. Health care professionals therefore need to pay attention to the language they use when recording health data in the Patient Data Repository. The aim of a patient portal is to make patients more involved in their own health and well-being, but this will not be achieved if patients do not understand the information provided in the service. In addition to simple language, the patient portal has to be simple enough for everyone to use. Of My Kanta users, approximately 16% expressed the desire to receive guidance on My Kanta use. This means that current information and guidance about My Kanta (eg, My Kanta pages, frequently-asked-question pages, and the online course) [26,42,43] need to be improved. As all nationally organized guidance is available only on the internet, face-to-face guidance about the use of My Kanta might also be needed. According to our previous study, main reasons for nonuse of My Kanta were the lack of need and tools [44]. In addition, some pharmacy customers had difficulties with My Kanta use and some were unfamiliar with the service. These results also underline the need for improving the guidance and information about the service.

### Strengths and Limitations

This study had several strengths. The patient portal studied here is nationwide and available to everyone living in Finland with internet access and an ID for electronic services. By distributing questionnaires at pharmacies to customers purchasing prescription medications, we reached a target population likely to need to use the patient portal. The study sample was large and included participants across Finland. We achieved our goal of reaching both users and nonusers of the patient portal, and these were distributed similarly by background information except for internet-related characteristics. The questionnaire did not include validated measures, but it was designed based on previous studies about patient portals [3-5,16,19,27-29,45], and some of the questions reported in this paper were based on previous surveys but had minor revisions [16,19,28,45]. The response rates for the questions reported in this paper were high (93%-100%), and, thus, it can be assumed that the questions were understandable.

This study also had limitations. Of the 3560 questionnaires delivered to the pharmacies, 694 questionnaires were not distributed. Most of the pharmacies distributed all the questionnaires, but a few pharmacies distributed one-half or less of the questionnaires delivered to them. We lacked the information on whether these pharmacies attempted to distribute these remaining questionnaires or if the pharmacies were not motivated to do so. The survey response rate was low. We had no information about who declined to participate in the study. As a result, the response rate may be even lower than reported. It was also lower than in studies conducted with the same method earlier in Finland (40%-44%) [16,46]. The trend in survey response rates has generally been declining in recent decades [16,46-49]. We do not have comparable statistics about pharmacy customers purchasing prescription medications in Finland, but, compared to customers receiving reimbursement for medication costs under the Health Insurance Scheme [50], the participants were older and more often women. This is also in line with trends in previous survey studies [16,46-48]. However, participants’ characteristics were similar to those in studies conducted previously with the same method [16,46].

### Conclusions

Pharmacy customers were satisfied with the nationwide patient portal. It was mostly used for browsing e-prescriptions and medical records. The usability of the service was mainly good, but users need to be better informed about data privacy and security issues as well as the guidance available for use of the portal.

## Appendix

Multimedia Appendix 1 A questionnaire.

Multimedia Appendix 2 Frequency of using My Kanta functions concerning electronic prescriptions and health data, and differences between groups.

Multimedia Appendix 3 Use of My Kanta functions concerning consents and limitations, and differences between groups.

## Abbreviations

e-prescription: electronic prescription
