# Supplementary material for: Pharmacy Customers’ Experiences of Use, Usability, and Satisfaction of a Nationwide Patient Portal: Survey Study
Source: J Med Internet Res. 2021 Jul 16;23(7):e25368. doi: 10.2196/25368 (PMC8325076; doi:10.2196/25368)
Supplement: Multimedia Appendix 1 [file jmir_v23i7e25368_app1.doc]

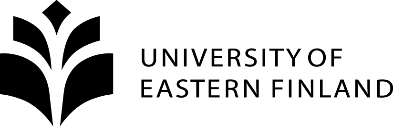
University of Eastern Finland

Faculty of Health Sciences

School of Pharmacy

Winter 2019

**SURVEY FOR PHARMACY CUSTOMERS REGARDING THE MY KANTA SERVICE**

Answer the questions by circling the number of the appropriate response option. If necessary, write your answer in the reserved space. For the success of the survey, it is important you answer all the questions that concern you.

Questions on **page 1** are meant for **all respondents**.

Questions on **pages 2 and 3** are meant for **those who use the My Kanta service**.

Questions on **page 4** are meant for **those who do not use the My Kanta service**.

**QUESTIONS ON THIS PAGE ARE MEANT FOR ALL RESPONDENTS**

1. **Your gender?**
2. Male
3. Female
4. **Your year of birth? _________**
5. **Your education?**
6. Basic education qualification
   (comprehensive school, middle school and primary school)
7. Vocational upper secondary qualification or vocational college diploma
8. Matriculation examination
9. Lower university degree
10. Higher university degree
11. **Area where you live?**
12. Southern Finland
13. Southwestern Finland
14. Western and Inland Finland
15. Eastern Finland
16. Northern Finland
17. Lapland
18. **Do you use the Internet (e.g. at home, at work, at your place of study, in a library)?**
19. Yes, daily or on several days a week
20. Yes, on one day a week or less often
21. I do not use the Internet
22. **Do you use the Internet to look up health-related information?**
23. Yes
24. No
25. **Do you have an ID for electronic services (e.g. online banking IDs, mobile ID, electronic identity card)?**
26. Yes
27. No
28. Don’t know
29. **At the moment, do you have any long-term illnesses diagnosed by a doctor?**
30. Yes
31. No
32. Don’t know
33. **Are you using any regular prescription medicines (e.g. medicines for high blood pressure or asthma) at the moment?**
34. Yes. How many? ______________
35. No
36. **Do you use the My Kanta service to browse your prescription and/or health information?**
37. Yes, I do

🡪 Go to **question 11 on page 2**

1. I have used it but I am not going to use it anymore

🡪 Go to **question 21 on page 4**

1. I have never used it

🡪 Go to **question 21 on page 4**

**QUESTIONS ON THE FOLLOWING TWO PAGES ARE MEANT FOR THOSE WHO USE
THE MY KANTA SERVICE**

1. **Have you used the following functions in the My Kanta service?**

|  | **Often** | **Sometimes** | **Rarely** | **Never** |
| --- | --- | --- | --- | --- |
| Browsing my prescription information | 1 | 2 | 3 | 4 |
| Requesting a prescription renewal | 1 | 2 | 3 | 4 |
| Printing out my prescription information | 1 | 2 | 3 | 4 |
| Browsing records of my healthcare visits | 1 | 2 | 3 | 4 |
| Browsing certificates and statements related to my health | 1 | 2 | 3 | 4 |
| Browsing my laboratory test and X-ray examination results | 1 | 2 | 3 | 4 |
| Browsing my disclosed information | 1 | 2 | 3 | 4 |
| Saving information on my well-being (e.g. weight, steps, activity) | 1 | 2 | 3 | 4 |

1. **Have you used the following functions in the My Kanta service?**

|  | **Often** | **Sometimes** | **Rarely** | **Never** | **I do not have dependants under 10 years of age** |
| --- | --- | --- | --- | --- | --- |
| Browsed the information of a dependant of mine under 10 years of age | 1 | 2 | 3 | 4 | 5 |
| Requested a prescription renewal for a dependant of mine under 10 years of age | 1 | 2 | 3 | 4 | 5 |

1. **Have you used the following functions in the My Kanta service?**

|  | **Yes** | **No** | **Don’t know** |
| --- | --- | --- | --- |
| Given consent for the disclosure of my health information | 1 | 2 | 3 |
| Limited the disclosure of my prescription information | 1 | 2 | 3 |
| Limited the disclosure of my health information | 1 | 2 | 3 |
| Declared my organ donation testament | 1 | 2 | 3 |
| Declared my living will | 1 | 2 | 3 |

1. **What do you think about the following statements? In each statement, circle the option you consider appropriate.**

|  | **Completely agree** | **Agree to some extent** | **Disagree to some extent** | **Completely disagree** | **Don’t know** |
| --- | --- | --- | --- | --- | --- |
| It is easy to log in in the My Kanta service | 1 | 2 | 3 | 4 | 5 |
| The view of the My Kanta service is clear | 1 | 2 | 3 | 4 | 5 |
| It is easy to monitor how much medicine is left on a prescription in the My Kanta service | 1 | 2 | 3 | 4 | 5 |
| It is easy to monitor expiry dates for prescriptions in the My Kanta service | 1 | 2 | 3 | 4 | 5 |
| It is easy to request a prescription renewal in the My Kanta service | 1 | 2 | 3 | 4 | 5 |
| It is easy to check in the My Kanta service whether my prescription has been renewed | 1 | 2 | 3 | 4 | 5 |
| It is easy to print out my prescription information in the My Kanta service | 1 | 2 | 3 | 4 | 5 |
| The My Kanta service provides a good overall picture of the medicines prescribed for me | 1 | 2 | 3 | 4 | 5 |
| In the My Kanta service, it is easy to see in which pharmacies and healthcare units my prescription information has been viewed | 1 | 2 | 3 | 4 | 5 |
|  | **Completely agree** | **Agree to some extent** | **Disagree to some extent** | **Completely disagree** | **Don’t know** |
| In the My Kanta service, it is easy to see in which healthcare units my health information has been processed | 1 | 2 | 3 | 4 | 5 |
| The My Kanta service is useful for monitoring my health information | 1 | 2 | 3 | 4 | 5 |
| It is easy to find the information I am looking for in the My Kanta service | 1 | 2 | 3 | 4 | 5 |
| The information recorded about me in the My Kanta service is easy to understand | 1 | 2 | 3 | 4 | 5 |
| The information recorded about me in the My Kanta service is correct | 1 | 2 | 3 | 4 | 5 |
| The My Kanta service works without problems | 1 | 2 | 3 | 4 | 5 |
| I fear that unauthorised persons may view my prescription and health information | 1 | 2 | 3 | 4 | 5 |
| I fear that my electronically saved prescription and health information may disappear | 1 | 2 | 3 | 4 | 5 |
| I would like to receive guidance in the use of the My Kanta service | 1 | 2 | 3 | 4 | 5 |

1. **What advantages or benefits has use of the My Kanta service provided to you?**

________________________________________________________________________________________________________________________________________________________________________________________

1. **What problems have you experienced when using the My Kanta service?**

________________________________________________________________________________________________________________________________________________________________________________________

1. **How could the My Kanta service be improved to make it easier for you to monitor and manage your medication and health information?**

________________________________________________________________________________________________________________________________________________________________________________________

1. **Nowadays, people can save information related to their well-being (e.g. information regarding measurements, lifestyle and activity) in the My Kanta service, and, in the future, people may, if they wish, share this information with healthcare professionals to support treatment decisions and diagnoses. What do you think about this?**
2. Necessary
3. Unnecessary 🡪 Go **to question 20**
4. Don’t know 🡪 Go **to question** **20**
5. **Which information related to your well-being would you like to save in the My Kanta service?**

________________________________________________________________________________________________________________________________________________________________________________________

1. **How satisfied are you with the My Kanta service as a whole? Circle the option you consider appropriate.**

| **Not satisfied at all** |  |  |  |  | **Very satisfied** |
| --- | --- | --- | --- | --- | --- |
| 1 | 2 | 3 | 4 | 5 | 6 |

**Thank you for answering.** **At the end of this survey, you can write any comments you may have about the survey and the My Kanta service.**

**QUESTIONS ON THIS PAGE ARE MEANT FOR THOSE WHO DO NOT USE
THE MY KANTA SERVICE**

1. **What are the main reasons why you do not use the My Kanta service?**

________________________________________________________________________________________________________________________________________________________________________________________

________________________________________________________________________________________________________________________________________________________________________________________

1. **How would you like to monitor your prescription and health information?**

________________________________________________________________________________________________________________________________________________________________________________________

________________________________________________________________________________________________________________________________________________________________________________________

**Thank you for answering**

**You can write any comments you may have about this survey and the My Kanta service below.**

________________________________________________________________________________________________________________________________________________________________________________________

____________________________________________________________________________________________

________________________________________________________________________________________________________________________________________________________________________________________

____________________________________________________________________________________________

________________________________________________________________________________________________________________________________________________________________________________________

________________________________________________________________________________________________________________________________________________________________________________________________________________________________________________________________________________________________________________________________________________________________________________________________________________________________________________________________________________________________________________________________________________________________________________________________________________________________________________________________________________________________________________________________________________________________

**Please check that you have answered all the questions that concern you.**

**Thank you**
