# Supplementary material for: Pharmacy Customers’ Experiences of Use, Usability, and Satisfaction of a Nationwide Patient Portal: Survey Study
Source: J Med Internet Res. 2021 Jul 16;23(7):e25368. doi: 10.2196/25368 (PMC8325076; doi:10.2196/25368)
Supplement: Multimedia Appendix 2 [file jmir_v23i7e25368_app2.doc]

Appendix 2. Frequency of using My Kanta functions concerning e-prescriptions and health data, and differences between groups.

|  | | **All, n (%)** | **Gender, n (%)** | | **Age (years), n (%)** | | | | **Education, n (%)** | | | **Internet use** | | **Internet use for searching health-related information** | | **Has any chronic disease diagnosed by a physician** | | **Current use of regular prescription medicines** | | |
| --- | --- | --- | --- | --- | --- | --- | --- | --- | --- | --- | --- | --- | --- | --- | --- | --- | --- | --- | --- | --- |
|  |  |  | **Female** | **Male** | **18‒34** | **35‒59** | **60‒74** | **75-** | **Basic education** | **Secondary education** | **University degree** | **Daily or on several days a week** | **Once a week or less often** | **Yes** | **No** | **Yes** | **No** | **0** | **1‒4** | **5-** |
|  | |  |  |  |  |  |  |  |  |  |  |  |  |  |  |  |  |  |  |  |
| **Browsing prescription information** | | | | | | | | | | | | | | | | | | | | |
|  | Often | 340  (42.4) | 239  (42.4) | 101  (42.6) | 18  (36.0) | 109  (46.6) | 164  (42.2) | 33  (32.4) | 55  (44.4) | 187  (43.2) | 98  (40.0) | 334  (44.0) | 6  (15.4) | 330  (43.7) | 10  (21.7) | 308  (46.2) | 25  (22.5) | 21  (25.0) | 191  (39.2) | 106  (55.5) |
|  | Sometimes | 348  (43.4) | 243  (43.1) | 104  (43.9) | 24  (48.0) | 98  (41.9) | 167  (42.9) | 52  (51.0) | 53  (42.7) | 180  (41.6) | 115  (46.9) | 324  (42.7) | 22  (56.4) | 326  (43.2) | 21  (45.7) | 275  (41.3) | 57  (51.4) | 44  (52.4) | 215  (44.1) | 75  (39.3) |
|  | Rarely | 93  (11.6) | 65  (11.5) | 28  (11.8) | 7  (14.0) | 23  (9.8) | 48  (12.3) | 11  (10.8) | 14  (11.3) | 53  (12.2) | 26  (10.6) | 84  (11.1) | 7  (17.9) | 83  (11.0) | 10  (21.7) | 68  (10.2) | 24  (21.6) | 14  (16.7) | 66  (13.6) | 9  (4.7) |
|  | Never | 21  (2.6) | 17  (3.0) | 4  (1.7) | 1  (2.0) | 4  (1.7) | 10  (2.6) | 6  (5.9) | 2  (1.6) | 13  (3.0) | 6  (2.4) | 17  (2.2) | 4  (10.3) | 16  (2.1) | 5  (10.9) | 15  (2.3) | 5  (4.5) | 5  (6.0) | 15  (3.1) | 1  (0.5) |
|  | *P*-value |  | .76 | | .25 | | | | .85 | | | <.001 | | <.001 | | <.001 | | <.001 | | |
| **Browsing records of healthcare visits** | | | | | | | | | | | | | | | | | | | | |
|  | Often | 306  (38.2) | 224  (39.6) | 82  (34.7) | 24  (48.0) | 94  (40.0) | 144  (37.4) | 31  (29.8) | 49  (38.9) | 171  (39.7) | 86  (35.1) | 298  (39.4) | 7  (17.5) | 298  (39.6) | 8  (16.7) | 271  (40.7) | 29  (26.1) | 23  (26.7) | 169  (34.6) | 94  (50.0) |
|  | Sometimes | 351  (43.8) | 249  (44.1) | 101  (42.8) | 18  (36.0) | 100  (42.6) | 172  (44.7) | 52  (50.0) | 49  (38.9) | 187  (43.4) | 115  (46.9) | 327  (43.2) | 22  (55.0) | 331  (44.0) | 20  (41.7) | 287  (43.1) | 56  (50.5) | 46  (53.5) | 229  (46.8) | 68  (36.2) |
|  | Rarely | 115  (14.3) | 75  (13.3) | 40  (16.9) | 6  (12.0) | 33  (14.0) | 53  (13.8) | 18  (17.3) | 24  (19.0) | 57  (13.2) | 34  (13.9) | 107  (14.1) | 6  (15.0) | 102  (13.5) | 13  (27.1) | 88  (13.2) | 20  (18.0) | 15  (17.4) | 71  (14.5) | 20  (10.6) |
|  | Never | 30  (3.7) | 17  (3.0) | 13  (5.5) | 2  (4.0) | 8  (3.4) | 16  (4.2) | 3  (2.9) | 4  (3.2) | 16  (3.7) | 10  (4.1) | 25  (3.3) | 5  (12.5) | 22  (2.9) | 7  (14.6) | 20  (3.0) | 6  (5.4) | 2  (2.3) | 20  (4.1) | 6  (3.2) |
|  | *P*-value |  | .14 | | .68 | | | | .59 | | | .002 | | <.001 | | .02 | | .003 | | |
| **Browsing laboratory test and X-ray examination results** | | | | | | | | | | | | | | | | | | | | |
|  | Often | 290  (36.1) | 203  (35.9) | 87  (36.7) | 20  (40.0) | 78  (33.2) | 145  (37.5) | 34  (33.0) | 52  (40.9) | 159  (36.6) | 79  (32.6) | 279  (36.7) | 9  (23.1) | 281  (37.2) | 9  (19.1) | 262  (39.2) | 24  (21.6) | 19  (22.4) | 164  (33.6) | 85  (44.3) |
|  | Sometimes | 325  (40.4) | 234  (41.3) | 91  (38.4) | 17  (34.0) | 106  (45.1) | 150  (38.8) | 44  (42.7) | 43  (33.9) | 182  (41.8) | 100  (41.3) | 307  (40.4) | 17  (43.6) | 309  (40.9) | 16  (34.0) | 260  (38.9) | 54  (48.6) | 40  (47.1) | 210  (43.0) | 69  (35.9) |
|  | Rarely | 107  (13.3) | 71  (12.5) | 35  (14.8) | 6  (12.0) | 23  (9.8) | 56  (14.5) | 17  (16.5) | 21  (16.5) | 56  (12.9) | 30  (12.4) | 98  (12.9) | 7  (17.9) | 97  (12.8) | 10  (21.3) | 85  (12.7) | 18  (16.2) | 13  (15.3) | 67  (13.7) | 21  (10.9) |
|  | Never | 82  (10.2) | 58  (10.2) | 24  (10.1) | 7  (14.0) | 28  (11.9) | 36  (9.3) | 8  (7.8) | 11  (8.7) | 38  (8.7) | 33  (13.6) | 76  (10.0) | 6  (15.4) | 69  (9.1) | 12  (25.5) | 62  (9.3) | 15  (13.5) | 13  (15.3) | 47  (9.6) | 17  (8.9) |
|  | *P*-value |  | .80 | | .43 | | | | .20 | | | .29 | | <.001 | | .005 | | .02 | | |
| **Browsing certificates and statements related to health** | | | | | | | | | | | | | | | | | | | | |
|  | Often | 194  (24.3) | 136  (24.3) | 58  (24.3) | 16  (32.0) | 57  (24.3) | 88  (22.9) | 23  (22.5) | 34  (27.0) | 107  (24.8) | 53  (21.8) | 186  (24.5) | 7  (18.9) | 187  (24.9) | 7  (14.9) | 176  (26.4) | 14  (12.8) | 11  (12.9) | 109  (22.3) | 62  (32.8) |
|  | Sometimes | 343  (42.9) | 240  (42.9) | 103  (43.1) | 16  (32.0) | 106  (45.1) | 163  (42.4) | 46  (45.1) | 51  (40.5) | 190  (44.1) | 102  (42.0) | 324  (42.7) | 18  (48.6) | 327  (43.5) | 16  (34.0) | 281  (42.2) | 53  (48.6) | 42  (49.4) | 207  (42.4) | 78  (41.3) |
|  | Rarely | 155  (19.4) | 105  (18.8) | 49  (20.5) | 10  (20.0) | 37  (15.7) | 86  (22.4) | 17  (16.7) | 30  (23.8) | 80  (18.6) | 45  (18.5) | 148  (19.5) | 5  (13.5) | 142  (18.9) | 13  (27.7) | 126  (18.9) | 20  (18.3) | 18  (21.2) | 102  (20.9) | 30  (15.9) |
|  | Never | 108  (13.5) | 79  (14.1) | 29  (12.1) | 8  (16.0) | 35  (14.9) | 47  (12.2) | 16  (15.7) | 11  (8.7) | 54  (12.5) | 43  (17.7) | 101  (13.3) | 7  (18.9) | 96  (12.8) | 11  (23.4) | 83  (12.5) | 22  (20.2) | 14  (16.5) | 70  (14.3) | 19  (10.1) |
|  | *P*-value |  | .86 | | .48 | | | | .20 | | | .53 | | .04 | | .007 | | .01 | | |
| **Requesting a prescription renewal** | | | | | | | | | | | | | | | | | | | | |
|  | Often | 268  (33.8) | 193  (34.6) | 75  (32.1) | 12  (24.0) | 74  (31.9) | 138  (35.9) | 31  (30.4) | 52  (43.0) | 149  (34.7) | 67  (27.6) | 257  (34.3) | 11  (27.5) | 253  (34.0) | 15  (31.9) | 252  (38.2) | 14  (12.7) | 11  (13.3) | 157  (32.6) | 85  (45.2) |
|  | Sometimes | 188  (23.7) | 122  (21.9) | 66  (28.2) | 12  (24.0) | 59  (25.4) | 85  (22.1) | 25  (24.5) | 27  (22.3) | 111  (25.9) | 50  (20.6) | 183  (24.4) | 5  (12.5) | 179  (24.0) | 8  (17.0) | 152  (23.1) | 26  (23.6) | 15  (18.1) | 115  (23.9) | 47  (25.0) |
|  | Rarely | 102  (12.9) | 71  (12.7) | 30  (12.8) | 11  (22.0) | 30  (12.9) | 47  (12.2) | 14  (13.7) | 14  (11.6) | 52  (12.1) | 36  (14.8) | 98  (13.1) | 3  (7.5) | 95  (12.8) | 7  (14.9) | 80  (12.1) | 18  (16.4) | 13  (15.7) | 65  (13.5) | 19  (10.1) |
|  | Never | 235  (29.6) | 172  (30.8) | 63  (26.9) | 15  (30.0) | 69  (29.7) | 114  (29.7) | 32  (31.4) | 28  (23.1) | 117  (27.3) | 90  (37.0) | 211  (28.2) | 21  (52.5) | 218  (29.3) | 17  (36.2) | 175  (26.6) | 52  (47.3) | 44  (53.0) | 145  (30.1) | 37  (19.7) |
|  | *P*-value |  | .27 | | .69 | | | | .01 | | | .01 | | .61 | | <.001 | | <.001 | | |
| **Browsing disclosed information** | | | | | | | | | | | | | | | | | | | | |
|  | Often | 48  (6.1) | 35  (6.4) | 13  (5.5) | 3  (6.0) | 13  (5.6) | 25  (6.6) | 3  (3.0) | 13  (10.7) | 26  (6.1) | 9  (3.7) | 48  (6.4) | 0  (0.0) | 48  (6.5) | 0  (0.0) | 43  (6.6) | 4  (3.7) | 4  (4.8) | 21  (4.4) | 15  (8.0) |
|  | Sometimes | 151  (19.2) | 102  (18.6) | 49  (20.7) | 6  (12.0) | 48  (20.6) | 80  (21.2) | 13  (13.0) | 18  (14.8) | 97  (22.9) | 36  (14.9) | 143  (19.1) | 8  (22.2) | 146  (19.7) | 5  (10.9) | 135  (20.6) | 13  (12.0) | 9  (10.8) | 92  (19.2) | 45  (23.9) |
|  | Rarely | 239  (30.4) | 161  (29.3) | 77  (32.5) | 21  (42.0) | 67  (28.8) | 116  (30.8) | 27  (27.0) | 35  (28.7) | 137  (32.4) | 67  (27.7) | 229  (30.6) | 8  (22.2) | 225  (30.4) | 14  (30.4) | 189  (28.9) | 41  (38.0) | 29  (34.9) | 148  (30.9) | 51  (27.1) |
|  | Never | 349  (44.3) | 251  (45.7) | 98  (41.4) | 20  (40.0) | 105  (45.1) | 156  (41.4) | 57  (57.0) | 56  (45.9) | 163  (38.5) | 130  (53.7) | 328  (43.9) | 20  (55.6) | 321  (43.4) | 27  (58.7) | 287  (43.9) | 50  (46.3) | 41  (49.4) | 218  (45.5) | 77  (41.0) |
|  | *P*-value |  | .60 | | .14 | | | | .001 | | | .23 | | .07 | | .06 | | .10 | | |
| **Printing out prescription information** | | | | | | | | | | | | | | | | | | | | |
|  | Often | 64  (8.4) | 39  (7.3) | 25  (11.0) | 2  (4.0) | 8  (3.4) | 35  (9.7) | 14  (14.6) | 15  (12.6) | 35  (8.5) | 14  (5.9) | 61  (8.4) | 3  (8.1) | 63  (8.8) | 1  (2.2) | 63  (9.9) | 1  (0.9) | 0  (0.0) | 33  (7.1) | 26  (14.4) |
|  | Sometimes | 120  (15.7) | 81  (15.1) | 39  (17.2) | 4  (8.0) | 35  (15.1) | 59  (16.3) | 18  (18.8) | 19  (16.0) | 60  (14.6) | 41  (17.4) | 117  (16.2) | 3  (8.1) | 113  (15.7) | 7  (15.2) | 108  (17.0) | 7  (6.5) | 2  (2.5) | 66  (14.1) | 44  (24.3) |
|  | Rarely | 143  (18.7) | 91  (16.9) | 52  (22.9) | 4  (8.0) | 45  (19.4) | 77  (21.3) | 12  (12.5) | 22  (18.5) | 73  (17.8) | 48  (20.3) | 139  (19.2) | 3  (8.1) | 137  (19.1) | 6  (13.0) | 125  (19.7) | 13  (12.1) | 7  (8.8) | 87  (18.6) | 45  (24.9) |
|  | Never | 438  (57.3) | 326  (60.7) | 111  (48.9) | 40  (80.0) | 144  (62.1) | 191  (52.8) | 52  (54.2) | 63  (52.9) | 242  (59.0) | 133  (56.4) | 407  (56.2) | 28  (75.7) | 405  (56.4) | 32  (69.6) | 338  (53.3) | 86  (80.4) | 71  (88.8) | 282  (60.3) | 66  (36.5) |
|  | *P*-value |  | .02 | | .001 | | | | .41 | | | .11 | | .22 | | <.001 | | <.001 | | |
