# Supplementary material for: Pharmacy Customers’ Experiences of Use, Usability, and Satisfaction of a Nationwide Patient Portal: Survey Study
Source: J Med Internet Res. 2021 Jul 16;23(7):e25368. doi: 10.2196/25368 (PMC8325076; doi:10.2196/25368)
Supplement: Multimedia Appendix 3 [file jmir_v23i7e25368_app3.doc]

Appendix 3. Use of My Kanta functions concerning consents and limitations, and differences between groups.

|  | | **All, n (%)** | **Gender, n (%)** | | **Age (years), n (%)** | | | | **Education, n (%)** | | | **Internet use, n (%)** | | **Internet use for searching health-related information, n (%)** | | **Has any chronic disease diagnosed by a physician, n (%)** | | **Current use of regular prescription medicines, n (%)** | | |
| --- | --- | --- | --- | --- | --- | --- | --- | --- | --- | --- | --- | --- | --- | --- | --- | --- | --- | --- | --- | --- |
|  |  |  | Female | Male | 18‒34 | 35‒59 | 60‒74 | 75- | Basic education | Secondary education | University degree | Daily or on several days a week | Once a week or less often | Yes | No | Yes | No | 0 | 1‒4 | 5- |
|  | |  |  |  |  |  |  |  |  |  |  |  |  |  |  |  |  |  |  |  |
| **Given consent for the disclosure of health information** | | | | | | | | | | | | | | | | | | | | |
|  | Yes | 584  (72.3) | 407  (71.8) | 176  (73.3) | 34  (68.0) | 176  (74.9) | 278  (71.6) | 73  (68.2) | 89  (70.6) | 319  (72.7) | 176  (72.4) | 562  (73.7) | 18  (45.0) | 556  (73.3) | 28  (58.3) | 490  (73.0) | 74  (66.1) | 56  (65.9) | 349  (70.9) | 152  (78.8) |
|  | No | 159  (19.7) | 113  (19.9) | 46  (19.2) | 4  (8.0) | 45  (19.1) | 81  (20.9) | 26  (24.3) | 28  (22.2) | 79  (18.0) | 52  (21.4) | 144  (18.9) | 15  (37.5) | 145  (19.1) | 13  (27.1) | 129  (19.2) | 26  (23.2) | 20  (23.5) | 100  (20.3) | 30  (15.5) |
|  | Do not know | 65  (8.0) | 47  (8.3) | 18  (7.5) | 12  (24.0) | 14  (6.0) | 29  (7.5) | 8  (7.5) | 9  (7.1) | 41  (9.3) | 15  (6.2) | 57  (7.5) | 7 (17.5) | 58  (7.6) | 7  (14.6) | 52  (7.7) | 12  (10.7) | 9  (10.6) | 43  (8.7) | 11  (5.7) |
|  | *P*-value |  | .89 | | .001 | | | | .47 | | | <.001 | | .06 | | .29 | | .17 | | |
| **Declared organ donation testament** | | | | | | | | | | | | | | | | | | | | |
|  | Yes | 151  (18.9) | 112  (19.9) | 39  (16.5) | 18  (36.0) | 60  (25.6) | 59  (15.2) | 9  (8.7) | 9  (7.4) | 80  (18.4) | 62  (25.3) | 148  (19.6) | 3  (7.3) | 144  (19.1) | 7  (14.9) | 124 (18.6) | 23  (20.9) | 17  (20.5) | 88  (17.9) | 37  (19.8) |
|  | No | 608  (76.0) | 423  (75.0) | 185  (78.4) | 30  (60.0) | 166  (70.9) | 304  (78.4) | 89  (86.4) | 101  (83.5) | 334  (77.0) | 173  (70.6) | 566  (75.1) | 38  (92.7) | 568  (75.5) | 39  (83.0) | 506  (76.1) | 83  (75.5) | 60  (72.3) | 377  (76.8) | 143  (76.5) |
|  | Do not know | 41  (5.1) | 29  (5.1) | 12  (5.1) | 2  (4.0) | 8  (3.4) | 25  (6.4) | 5  (4.9) | 11  (9.1) | 20  (4.6) | 10  (4.1) | 40  (5.3) | 0  (0.0) | 40  (5.3) | 1  (2.1) | 35  (5.3) | 4  (3.6) | 6  (7.2) | 26  (5.3) | 7  (3.7) |
|  | *P*-value |  | .54 | | <.001 | | | | <.001 | | | .03 | | .44 | | .69 | | .73 | | |
| **Declared living will** | | | | | | | | | | | | | | | | | | | | |
|  | Yes | 95  (11.8) | 67  (11.9) | 28  (11.8) | 7  (14.0) | 31  (13.2) | 39  (10.1) | 14  (13.3) | 12  (9.8) | 45  (10.3) | 38  (15.4) | 89  (11.7) | 6  (15.0) | 90  (11.9) | 5  (10.6) | 80  (12.0) | 14  (12.8) | 8  (9.8) | 54  (11.0) | 27  (14.2) |
|  | No | 666  (82.9) | 470  (83.2) | 195  (82.3) | 38  (76.0) | 193  (82.1) | 329  (84.8) | 87  (82.9) | 102  (83.6) | 363  (83.4) | 201  (81.7) | 629  (83.0) | 33  (82.5) | 624  (82.6) | 41  (87.2) | 556  (83.1) | 91  (83.5) | 70  (85.4) | 410  (83.3) | 154  (81.1) |
|  | Do not know | 42  (5.2) | 28  (5.0) | 14  (5.9) | 5  (10.0) | 11  (4.7) | 20  (5.2) | 4  (3.8) | 8  (6.6) | 27  (6.2) | 7  (2.8) | 40  (5.3) | 1  (2.5) | 41  (5.4) | 1  (2.1) | 33  (4.9) | 4  (3.7) | 4  (4.9) | 28  (5.7) | 9  (4.7) |
|  | *P*-value |  | .86 | | .54 | | | | .10 | | | .70 | | .58 | | .83 | | .75 | | |
| **Limited the disclosure of health information** | | | | | | | | | | | | | | | | | | | | |
|  | Yes | 45  (5.6) | 32  (5.7) | 13  (5.6) | 4  (8.0) | 16  (6.8) | 16  (4.2) | 7  (6.9) | 8  (6.7) | 26  (6.0) | 11  (4.5) | 42  (5.6) | 3  (7.5) | 43  (5.7) | 2  (4.3) | 40  (6.0) | 4  (3.6) | 5  (6.0) | 25  (5.1) | 13 (6.9) |
|  | No | 693  (87.0) | 487  (86.3) | 205  (88.4) | 37  (74.0) | 207  (88.1) | 343  (89.1) | 85  (83.3) | 102  (85.0) | 370  (85.8) | 221  (89.8) | 656  (87.1) | 33  (82.5) | 650  (86.8) | 42  (89.4) | 572  (86.4) | 101  (91.8) | 74  (89.2) | 422  (86.7) | 167  (88.4) |
|  | Do not know | 59  (7.4) | 45  (8.0) | 14  (6.0) | 9  (18.0) | 12  (5.1) | 26  (6.8) | 10  (9.8) | 10  (8.3) | 35  (8.1) | 14  (5.7) | 55  (7.3) | 4  (10.0) | 56  (7.5) | 3  (6.4) | 50  (7.6) | 5  (4.5) | 4  (4.8) | 40  (8.2) | 9  (4.8) |
|  | *P*-value |  | .63 | | .02 | | | | .61 | | | .53 | | >.99 | | .29 | | .44 | | |
| **Limited the disclosure of prescription information** | | | | | | | | | | | | | | | | | | | | |
|  | Yes | 26  (3.3) | 14  (2.5) | 12  (5.1) | 3  (6.0) | 9  (3.8) | 11  (2.9) | 3  (2.9) | 3  (2.5) | 14  (3.2) | 9  (3.7) | 24  (3.2) | 0  (0.0) | 24  (3.2) | 2  (4.2) | 24  (3.6) | 2  (1.8) | 4  (4.8) | 13  (2.7) | 9  (4.8) |
|  | No | 710  (88.9) | 503  (89.3) | 206  (87.7) | 40  (80.0) | 217  (92.3) | 342  (88.8) | 89  (85.6) | 105  (89.0) | 382  (87.8) | 223  (90.7) | 673  (89.3) | 33  (84.6) | 666  (88.8) | 43  (89.6) | 589  (88.7) | 100  (90.9) | 72  (85.7) | 437  (89.5) | 170  (89.9) |
|  | Do not know | 63  (7.9) | 46  (8.2) | 17  (7.2) | 7  (14.0) | 9  (3.8) | 32  (8.3) | 12  (11.5) | 10  (8.5) | 39  (9.0) | 14  (5.7) | 57  (7.6) | 6  (15.4) | 60  (8.0) | 3  (6.3) | 51  (7.7) | 8  (7.3) | 8  (9.5) | 38  (7.8) | 10  (5.3) |
|  | *P*-value |  | .16 | | .04 | | | | .62 | | | .16 | | .86 | | .61 | | .39 | | |
